# Establishment of a Rapid and Efficient Method for the Detection of Avian Reovirus Based on RT-RAA-CRISPR/Cas12a Technology

**DOI:** 10.3390/ani15202994

**Published:** 2025-10-16

**Authors:** Qi Zheng, Zhiyuan Lu, Huahua Chen, Muzi Li, Haoyi Zhang, Ziqiang Cheng, Jianzhu Liu

**Affiliations:** 1 College of Veterinary Medicine, Shandong Agricultural University, Tai’an 271018, China; 18562201229@163.com (Q.Z.); 19860939156@163.com (H.C.); limz395430@163.com (M.L.); 2 Shandong Provincial Key Laboratory of Zoonoses, Shandong Agricultural University, Tai’an 271018, China; 2022110423@sdau.edu.cn (Z.L.); zhanghaoyi45@163.com (H.Z.)

**Keywords:** RT-RAA, CRISPR/CAS12a, avian reovirus (ARV), visual detection

## Abstract

**Simple Summary:**

Avian reovirus (ARV) is a significant arthrogenic virus in the poultry industry that is capable of infecting various avian species and leads to substantial economic losses. In this study, we developed for the first time a rapid detection system for ARV based on reverse transcription–recombinase-aided amplification (RT-RAA) combined with clustered regularly interspaced short palindromic repeats (CRISPR) technology. Using the recombinant plasmid pMD18T-ARVS1 as a template, the assay achieved high sensitivity at 37 °C within 40 min, with a detection limit as low as 1 copy/μL—10 times more sensitive than a comparable qPCR method. Specificity testing demonstrated that the RT-RAA-CRISPR/Cas12a assay does not cross-react with other common avian viruses that significantly impact poultry farming. Furthermore, the results can be visualized under blue light, making this accurate and portable detection method highly promising for ARV control in the poultry industry.

**Abstract:**

Avian reovirus (ARV), a highly pathogenic agent in poultry, causes severe economic losses through immunosuppression and secondary infections. Traditional diagnostic methods like reverse transcription quantitative PCR (RT-qPCR) and enzyme-linked immunosorbent assay (ELISA) face limitations in resource-limited settings due to equipment dependency and prolonged processing. To address this, we developed a rapid, portable detection method integrating reverse transcription–recombinase-aided amplification (RT-RAA) with CRISPR/Cas12a. By targeting the conserved P17-coding region of the ARV S1 gene, this assay amplifies viral RNA isothermally (37 °C) within 20 min, followed by Cas12a-mediated collateral cleavage of fluorescent or lateral flow reporters for visual readout. The method achieved a sensitivity of 1 copy/μL, surpassing RT-qPCR (10 copies/μL), and completed detection in 40 min. Specificity tests against non-target pathogens confirmed zero cross-reactivity. Utilizing a portable incubator and low-cost visual tools, this platform eliminates reliance on thermocyclers and skilled personnel. Its field-deployable design enables on-site diagnosis, facilitating early ARV detection to mitigate outbreaks and economic losses in poultry farming. This study provides a paradigm shift in avian pathogen surveillance, combining speed, sensitivity, and accessibility for global agricultural and public health applications.

## 1. Introduction

ARV, a double-stranded RNA member of the genus Orthoreovirus, represents a serious and global challenge to the poultry industry, jeopardizing flock health and economic viability [1]. With high genetic variability and multiple transmission modes, ARV is responsible for severe diseases in poultry—such as chickens, ducks, and geese—manifesting as arthritis, tenosynovitis, immunosuppression, and death [2,3,4,5,6,7]. The economic impact of ARV arises from direct mortality, damage to immune-related organs (e.g., bursa, spleen, and thymus), and secondary infections facilitated by immunosuppression, collectively resulting in significant economic burdens [8,9,10]. As the world’s largest poultry producer, China, with an annual poultry output of 17.34 billion birds in 2024—accounting for 16.8% of global poultry meat production according to the National Bureau of Statistics—is a major player in the worldwide industry. Given this scale, diseases like ARV, which cause significant economic losses through immunosuppression and secondary infections, have profound implications not only for China’s national economy but also for global poultry production. While comprehensive monetary estimates of ARV’s total global and national economic impact are difficult to ascertain due to varied reporting and indirect losses, its contribution to reduced productivity, increased mortality, and heightened disease susceptibility represents a substantial financial burden on both China’s extensive poultry sector and the international industry. Consequently, early and accurate detection of ARV is critical to mitigating its impact on both animal welfare and agricultural economies.

ARV particles are non-enveloped, icosahedral viruses with a double-capsid symmetry and a double-stranded RNA genome. The genome comprises 10 segments, which are classified based on their electrophoretic mobility in SDS-PAGE into three size classes: large (L1–L3), medium (M1–M3), and small (S1–S4) [11]. The avian reovirus S1 gene segment harbors a unique tricistronic arrangement, containing three distinct but partially overlapping open reading frames (ORFs) that encode the p10, p17/p18, and σC proteins, with the σC protein exhibiting the highest degree of variability [9,12,13,14,15,16]. The study of translation initiation mechanisms and functional properties of the proteins encoded by the three S1 ORFs is essential to discover highly specific marker genes for ARV detection [17]. However, the P17 protein is highly conserved, which makes it an ideal candidate for ARV detection and diagnosis [18]. Therefore, utilizing the highly conserved P17 protein as a target simplifies assay design and promises high specificity and reliability, facilitating the development of broad-spectrum ARV detection methods.

Traditional diagnostic methods for ARV primarily rely on serological assays (e.g., ELISA) and molecular techniques such as reverse transcription quantitative PCR (RT-qPCR). Although RT-qPCR is widely recognized for its high sensitivity and specificity [19,20], its dependency on thermocyclers, skilled personnel, and prolonged turnaround times significantly limits its utility in resource-constrained settings. Similarly, while an enzyme-linked immunosorbent assay (ELISA) is advantageous for large-scale screening, it is prone to cross-reactivity risks and prolonged incubation steps. Additionally, the sensitivity and specificity of ELISA results are critically dependent on antigen selection, requiring meticulous optimization to mitigate diagnostic inconsistencies [21,22]. Isothermal amplification methods like loop-mediated isothermal amplification (LAMP) offer equipment-free alternatives but require complex primer designs and lack multiplexing capabilities [23]. These limitations underscore an urgent need for a rapid, portable, and ultrasensitive detection platform capable of on-site deployment.

Recent advances in CRISPR-Cas systems have revolutionized nucleic acid diagnostics by enabling sequence-specific recognition and collateral cleavage activity [24]. Cas12a, a CRISPR-associated nuclease, exhibits trans-cleavage activity toward single-stranded DNA (ssDNA) upon target recognition [25], enabling signal amplification through fluorescence or lateral flow readouts. When integrated with isothermal amplification techniques such as recombinase-aided amplification (RAA) [26,27,28,29], this system not only retains the advantages of recombinase polymerase amplification (RPA) but also enhances cost-effectiveness, particularly in resource-limited settings [30]. Notably, reverse transcription–recombinase-aided amplification (RT-RAA) eliminates the need for thermal cycling by enabling target nucleic acid amplification under isothermal conditions (37–42 °C) within 20–30 min [31]. This approach thereby achieves highly sensitive and specific detection of target genes [32,33], offering a streamlined alternative to conventional PCR-based methods while maintaining compatibility with resource-limited settings.

In this study, we combined CRISPR/Cas12a with RAA technology to rapidly amplify the key gene S1 for ARV detection [34], for efficient diagnostics. By integrating the rapid amplification capability of RT-RAA with the collateral cleavage activity of Cas12a, this system enables visual detection through portable blue-light fluorescence analyzers or lateral flow strips, thereby achieving equipment-free, on-site diagnostics in resource-limited environments. Compared to RT-qPCR, this method reduces total assay time by 50% and achieves a 10-fold lower detection limit (1 copy/μL), as validated through cross-reactivity studies against non-target avian pathogens. By leveraging cost-effective, field-deployable instrumentation, this platform bridges the diagnostic gap in low-resource poultry farms, offering a paradigm shift in ARV surveillance and outbreak management.

## 2. Materials and Methods

### 2.1. Sample Preparation

All experimental viral strains were cryopreserved at −80 °C in the Pathogen Bank of the College of Veterinary Medicine at Shandong Agricultural University. Viral RNA was isolated from viral samples using the TIANamp Virus DNA/RNA Kit (TianGen Biotech Co., Ltd., Beijing, China) according to the manufacturer’s instructions. RNA templates were reverse-transcribed into complementary DNA (cDNA) using the All-in-One First-Strand Synthesis MasterMix (Yugong Biotech Co., Ltd., Lianyungang, China). Target fragments were amplified by PCR using the full-length ARV S1 gene sequence as a template with the S1-specific primers F (5′-GGTGCGACTGCTGTATTTGGTAAC-3′) and R (5′-AATGGAACGATAGCGTGTGGG-3′) (Table 1). The PCR products were separated by agarose gel electrophoresis, excised, and purified using the E.Z.N.A.^®^ Gel Extraction Kit (Omega Bio-Tek, Norcross, GA, USA). The purified products were cloned into the pMD™-18T Vector (Takara Biomedical Technology Co., Ltd., Beijing, China) according to the manufacturer’s protocol, resulting in the recombinant plasmid pMD18T-ARVS1. After transformation into JM109 chemically competent cells, plasmid DNA was purified with the E.Z.N.A.^®^ Plasmid Mini Kit I (Omega Bio-Tek, Norcross, GA, USA). Constructs verified by PCR were quantified using a BioTek Epoch spectrophotometer (BioTek Instruments, Winooski, VT, USA) and stored at –20 °C for further use.

### 2.2. Design and Optimization of Primers and crRNAs

**Figure 1:**
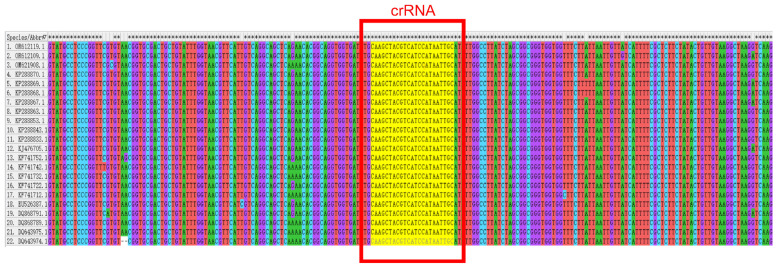
Comparison of different ARV-S1 sequences and crRNA target sites.

We designed all CRISPR RNAs (crRNA) through the Ezassay platform and prioritized top-ranked candidates for preliminary validation. Twenty-two full-length ARV-S1 sequences were retrieved from the NCBI database (Accessed on 23 April 2025 http://www.ncbi.nlm.nih.gov/) and aligned using MEGA software to determine conserved regions (Figure 1). The crRNA is designed to be complementary to a specific target sequence, enabling it to precisely recognize and bind to the corresponding DNA region—namely, the sequence boxed in Table 2. The optimal crRNA sequence was subsequently selected for downstream applications (Table 2). In order to get the best combination of RAA primers and crRNA [35].

Four candidate primers were designed according to the RAA primer design principle. Using the pMD18T-ARVS1 plasmid DNA as the template. The RT-RAA Nucleic Acid Amplification Kit (Basic Type) (Huicheng Biotechnology Co., Ltd., Shanghai, China) reaction was performed in a final volume of 25 μL according to the manufacturer’s protocol. Four sets of RT-RAA primers (10μM) (Table 1) were systematically evaluated, with each primer set tested in triplicate using ddH_2_O as the no-template control (NTC). Optimal primer pairs were subsequently identified through agarose gel electrophoresis analysis (Table 1). Given LbCas12a’s preference for cleaving thymine-rich single-stranded DNA (ssDNA), a TTATT ssDNA reporter was engineered as the detection substrate, modified with a 5′-FAM fluorophore and 3′-BHQ1/biotin quencher to enable real-time fluorescence signal monitoring (synthesized and purified by Sangon Biotech (Shanghai) Co., Ltd. (Shanghai, China)) (Table 2). Simultaneously, conventional S1-PCR primers targeting the CDS region were developed through PubMed-derived sequence analysis, ensuring coverage of critical genomic fragments (Table 1).

### 2.3. LbCas12a Protein Extraction and Purification

The pMBP-LbCas12a plasmid (NovoPro Biotechnology Co., Ltd., Shanghai, China) was transformed into Escherichia coli BL21 competent cells (AngYu Biotechnologies Co., Ltd., Shanghai, China) and plated on kanamycin-supplemented (50 μg/mL) LB agar plates. After overnight incubation at 37 °C, single colonies were selected and inoculated into 100 mL of LB medium. The culture was grown aerobically at 37 °C until the exponential growth phase was reached. Protein expression was then induced by adding isopropyl β-D-1-thiogalactopyranoside (IPTG; Yeasen Biotechnology Co., Ltd., Shanghai, China) to a final concentration of 1 mM, followed by incubation for 4–5 h. Bacterial cells were harvested by centrifugation at 5000× *g* for 5 min at room temperature. The LbCas12a protein was purified under native conditions using a His-tag Protein Purification Kit (Beyotime Biotechnology Co., Ltd., Shanghai, China). The purity of the eluted protein was evaluated by SDS-PAGE with Coomassie brilliant blue staining, and concentration was determined using a BCA Protein Assay Kit (Beyotime, Shanghai, China). Purified LbCas12a protein was aliquoted and stored at –80 °C until further use.

### 2.4. Verification of LbCas12a Protein Cleavage Activity

The validated optimal RT-RAA primers were used to amplify the pMD18T-ARVS1 standard plasmid in a 50 μL reaction volume. The complete reaction mixture for the positive control group consisted of ddH_2_O, 5 μL of 10× NEBuffer, 50 nM crRNA, 50 nM LbCas12a protein, and 500 nM fluorescent-quenched (FQ) reporter. Experimental groups were systematically designed by omitting individual key components: one without the Cas12a protein, one without the FQ reporter, one without the RAA amplicon, and one without crRNA. All omitted components were replaced with an equal volume of ddH_2_O to maintain consistent reaction volumes across groups. All reactions were incubated at 37 °C for 20 min, and fluorescence signals were detected using a blue-light gel imaging system (HTJY, Beijing, China).

### 2.5. Optimization of RT-RAA Detection Based on CRISPR/Cas12a

The Cas12a-mediated fluorescence detection reaction was standardized to a final volume of 50 µL, containing 250 nM LbCas12a, 250 nM crRNA, 500 nM fluorescent-quenched (FQ) reporter, 2 µL of RT-RAA amplicon, and 5 µL of 10× NEBuffer™ 2.1 (New England Biolabs Co., Ltd., Beijing, China). To optimize detection performance, key parameters including the amplification temperature (37 °C) and duration (20 min) were first established. Subsequently, a systematic optimization of component concentrations was conducted using an orthogonal experimental design: the FQ reporter was tested at concentrations ranging from 100 to 500 nM (in increments of 100 nM), while LbCas12a protein and crRNA were evaluated across a range of 25–500 nM using twofold serial dilutions. Real-time fluorescence intensity was monitored at 5 min intervals using an LC96 real-time PCR system. Fluorescence results were further validated using a blue-light gel imaging system, and optimal component concentrations were determined based on both kinetic and endpoint analyses.

For the Cas12a-mediated lateral flow assay, the reaction was similarly assembled in a 50 µL system, comprising 2 µL of RT-RAA amplicon, 250 nM LbCas12a, 250 nM crRNA, 5 µL of 10× NEBuffer™ 2.1, and 500 nM biotin- and FAM-modified lateral flow (LF) probe. After incubation at 37 °C, lateral flow test strips (Tolo Biotech Co., Ltd., Shanghai, China) were inserted into the reaction tubes at 2, 5, 10, 15, 20, and 25 min. The strips were immersed until the solution migrated through the entire reading area (typically 1–2 min). Results were interpreted visually: the appearance of both a control line (C-line) and a test line (T-line) indicated a positive result, while only the C-line represented a negative outcome.

### 2.6. Analytical Specificity and Sensitivity of the RT-RAA-CRISPR/Cas12a Detection Platform

To evaluate the specificity of the RT-RAA–CRISPR/Cas12a detection system, nucleic acids from several representative avian pathogens—including Reticuloendotheliosis virus (REV), Infectious Bursal Disease virus (IBDV), Avian Leukosis virus (ALV), Chicken Astrovirus (CAstV), and Chicken Anemia virus (CAV)—were analyzed under optimized reaction conditions. DEPC-treated water was used as the negative control, and the recombinant plasmid pMD18T-ARVS1 served as the positive control. Each viral nucleic acid sample was tested in at least three independent replicates to ensure rigorous validation of assay specificity.

For the sensitivity assay, the purified recombinant plasmid pMD18T-ARVS1 was used as the template. The plasmid concentration was quantified using a BioTek Epoch spectrophotometer, and 10-fold serial dilutions were prepared in TE buffer to obtain concentrations ranging from 10^0^ to 10^5^ copies/μL. Each dilution was tested in triplicate.

RT-qPCR amplification was carried out in a 20 μL reaction mixture containing: 6.2 μL of ddH_2_O, 10 μL of SYBR Green Pro Taq HS Premix (Accurate Biology Co., Ltd., Changsha, China), 3 μL of diluted pMD18T-ARVS1 template, 0.4 μL of qPCR-F forward primer, and 0.4 μL of qPCR-R reverse primer (Table 2). The amplification was performed under the following conditions (Table 3).

### 2.7. Statistical Analysis

The data are presented as mean ± SD. Statistical significance was determined using one-way analysis of variance (ANOVA) in GraphPad Prism software (version 10.1.2). A *p*-value less than 0.05 was considered statistically significant.

## 3. Results

### 3.1. RT-RAA Optimal Primer Screening

**Figure 2:**
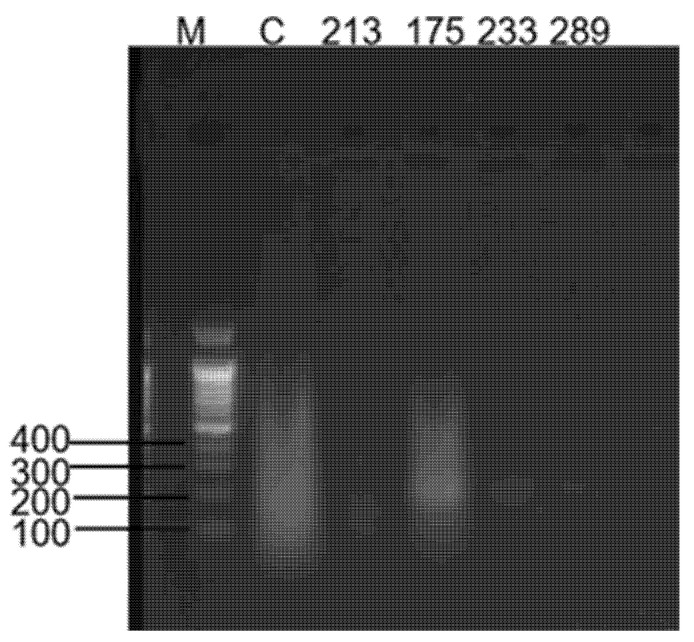
Optimal screening of four groups of RT-RAA primers. From left to right, the lanes represent: C (negative control), 213 (primers amplifying a 213 bp product), 175 (primers amplifying a 175 bp product), 233 (primers amplifying a 233 bp product), and 289 (primers amplifying a 289 bp product).

As shown in Figure 2, four RT-RAA primer sets (Table 2) were evaluated using pMD18T-ARVS1 as a template. Agarose gel electrophoresis analysis indicated that the RT-RAA2 primer pair demonstrated superior amplification efficiency compared to the other candidate sets.

### 3.2. Cas12a Protein Induction, Purification, and Activity Validation

**Figure 3:**
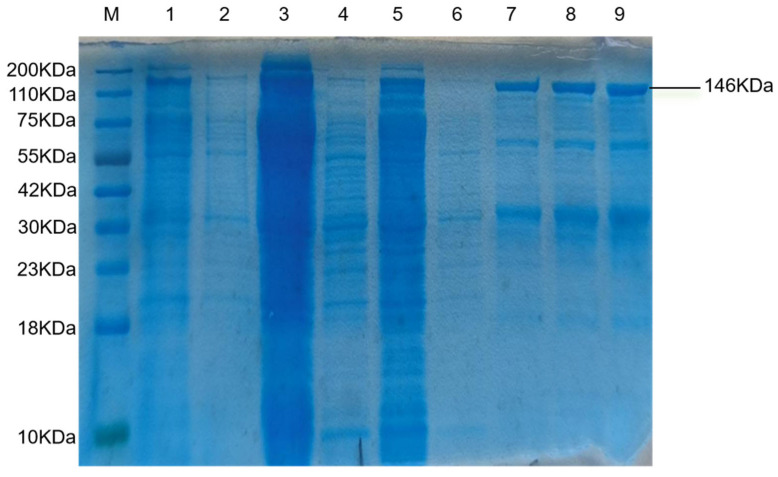
Activity verification of purified Cas12a protein. 1. IPTG induces bacterial fluid; 2. No lPTG-induced bacterial solution; 3. Through-fluid; 4. Washing liquid 1; 5. Washing solution 2; 6. Washing solution 3; 7. Purified protein 2; 8. Purified protein 3; 9. Purified protein 4.

Recombinant plasmid-containing E. coli BL21 cells were induced with 1 mM IPTG. Following purification, SDS-PAGE analysis with Coomassie blue staining revealed a distinct band at approximately 146 kDa (Figure 3), confirming successful expression and purification of the recombinant Cas12a protein.

**Figure 4:**
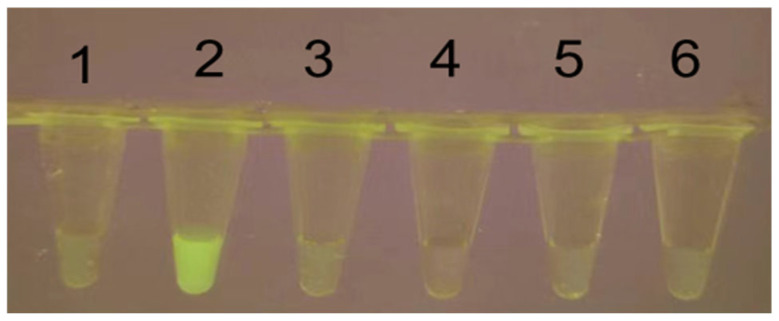
Verification of Cas12a activity with fluorescence. 1. ddH2O; 2. RAA amplicon+Cas12a+Fluorescent quencher (FQ)+crRNA; 3. RAA amplicont+FQ+crRNA; 4. Cas12a+FQ+crRNA; 5. RAA amplicon+Cas12a+crRNA; 6. RAA amplicon+Cas12a+FQ.

The nuclease activity of LbCas12a is triggered upon formation of a ternary complex with crRNA and double-stranded DNA (dsDNA). To verify this activation mechanism, a series of systematically designed negative controls was implemented by individually omitting key reaction components (LbCas12a, crRNA, or dsDNA amplicon). Fluorescence emission was observed exclusively in the complete reaction system (Tube 2) under blue-light transillumination (Figure 4), confirming the target-dependent activation of Cas12a cleavage activity.

### 3.3. CRISPR/Cas12a-Enhanced RT-RAA for Optimized Nucleic Acid Detection

**Figure 5:**
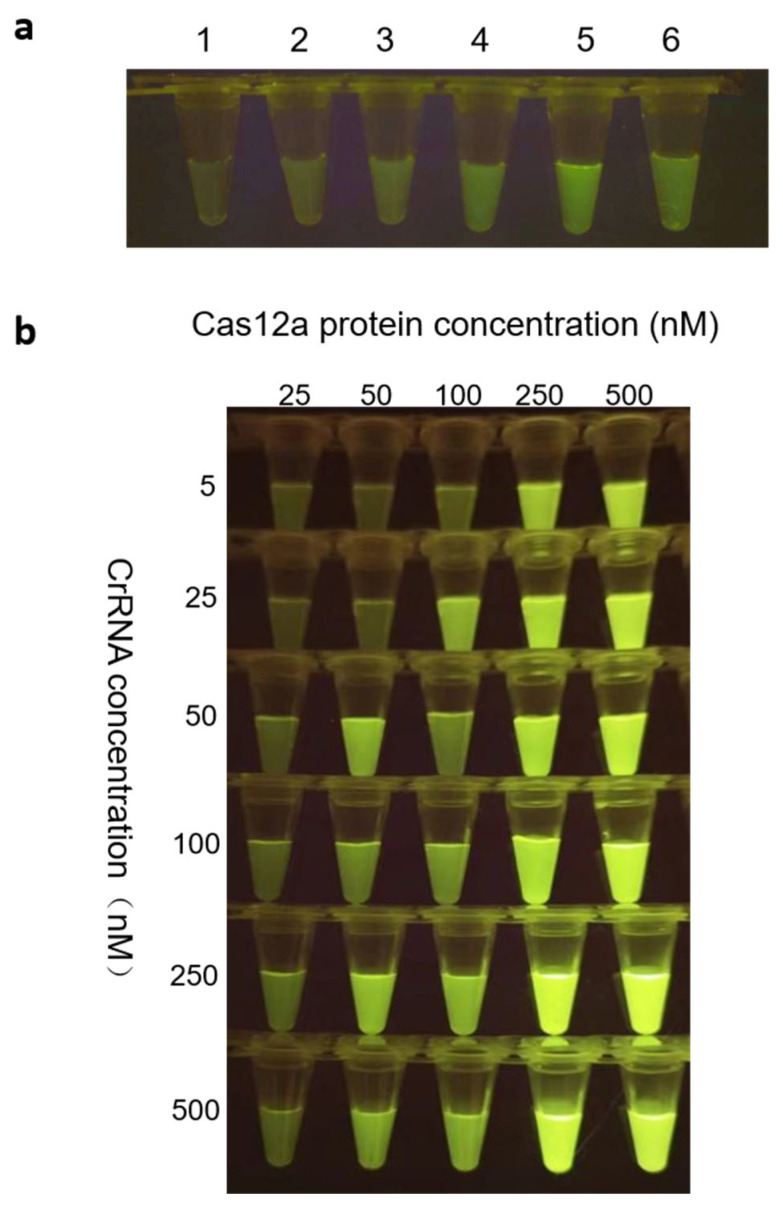
Evaluation of probe concentration and the optimal combination of Cas12a protein and crRNA. (**a**) Screening optimal probe concentrations of 1. 100 nM, 2. 200 nM, 3. 300 nM, 4. 400 nM, 5. 500 nM, and 6. 600 nM. (**b**) Screening of the optimal combination of Cas12a protein and crRNA.

To optimize detection efficiency, a comprehensive assessment of all relevant reaction variables was conducted. All experiments were performed in triplicate. Fluorescence intensity was quantified using a blue-light gel imaging system across a range of FQ reporter concentrations. The results indicated that 500 nM FQ yielded the highest fluorescence signal (Figure 5a), and this concentration was selected as optimal for all subsequent assays.

The stoichiometric ratio between crRNA and LbCas12a was systematically evaluated using two complementary methods: qualitative analysis via band intensity under blue-light imaging (Figure 5b) and quantitative fluorescence measurement using an LC96 real-time PCR system. All replicate data are presented as mean ± standard deviation (Table 4). Both methods consistently supported 250 nM as the ideal equimolar concentration for each component.

**Figure 6:**
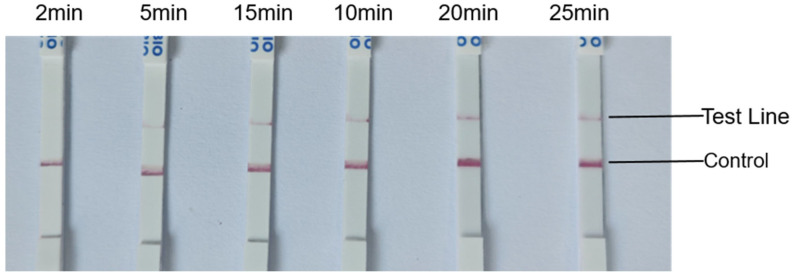
Optimization of Cas12a reaction time for evaluating the sensitivity of the lateral chromatography method.

Furthermore, the influence of Cas12a reaction time on lateral flow strip results was evaluated. As shown in Figure 6, a clear test line was observed within 20 min of Cas12a/crRNA activation. Thus, a 20 min reaction time was chosen for all subsequent Cas12a-mediated detection steps.

### 3.4. Specificity of RT-RAA-CRISPR/Cas12a

**Figure 7:**
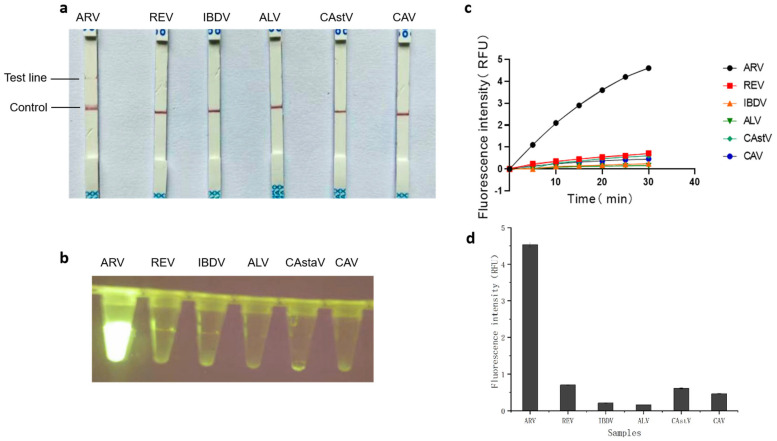
Specificity assessment of RT-RAA-CRISPR/Cas12a-FQ and RT-RAA-CRISPR/Cas12a-LF in ARV detection. (**a**) Plot of test strip results for RT-RAA-CRISPR/Cas12a-LF assay. (**b**) Fluorescence schematic of the RT-RAA-CRISPR/Cas12a-FQ assay. (**c**) Fluorescence amplification of the fluorescence assay of the RT-RAA/CRISPR system (**d**) Error analysis for ARV-specific assays.

The specificity of the RT-RAA–CRISPR/Cas12a assay was evaluated using genomic templates from several non-target avian pathogens, including REV, IBDV, ALV, CASTV, and CAV, under optimized reaction conditions. As shown in Figure 7b,d, a strong fluorescence signal was detected only in reactions containing ARV target sequences, with statistically significant differences (*p* < 0.01) compared to non-target pathogens. Corresponding results from the Cas12a/crRNA-based lateral flow assay showed clear test bands exclusively in ARV-positive samples (Figure 7a,c). Together, these findings confirm the high specificity of both fluorescent and lateral flow detection modalities for ARV, with no cross-reactivity observed against any non-target viruses.

### 3.5. Sensitivity Assay of the ARV-RT-RAA-CRISPR/Cas12a Detection System

**Figure 8:**
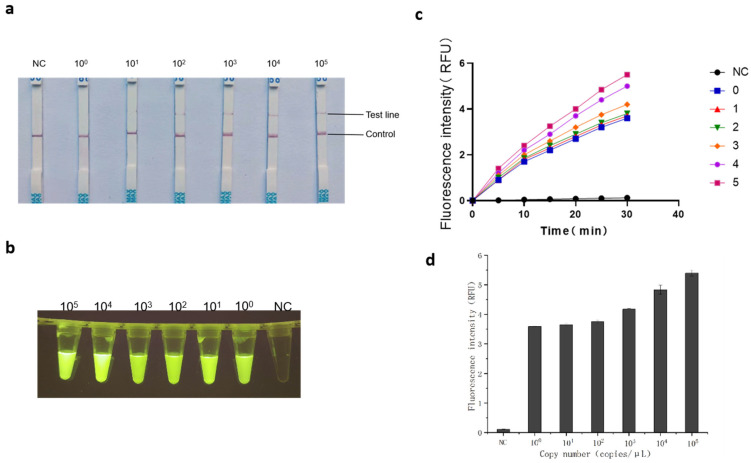
Evaluation of the Sensitivity of RT-RAA-CRISPR/Cas12a-FQ and RT-RAA-CRISPR/Cas12a-LF in ARV Detection. (**a**) Plot of test strip results for RT-RAA-CRISPR/Cas12a-LF assay. (**b**) Fluorescence schematic of the RT-RAA-CRISPR/Cas12a-FQ assay. (**c**) Fluorescence amplification of the fluorescence assay of the RT-RAA/CRISPR system. (**d**) Error analysis of ARV sensitivity detection. “NC” stands for negative control.

**Figure 9:**
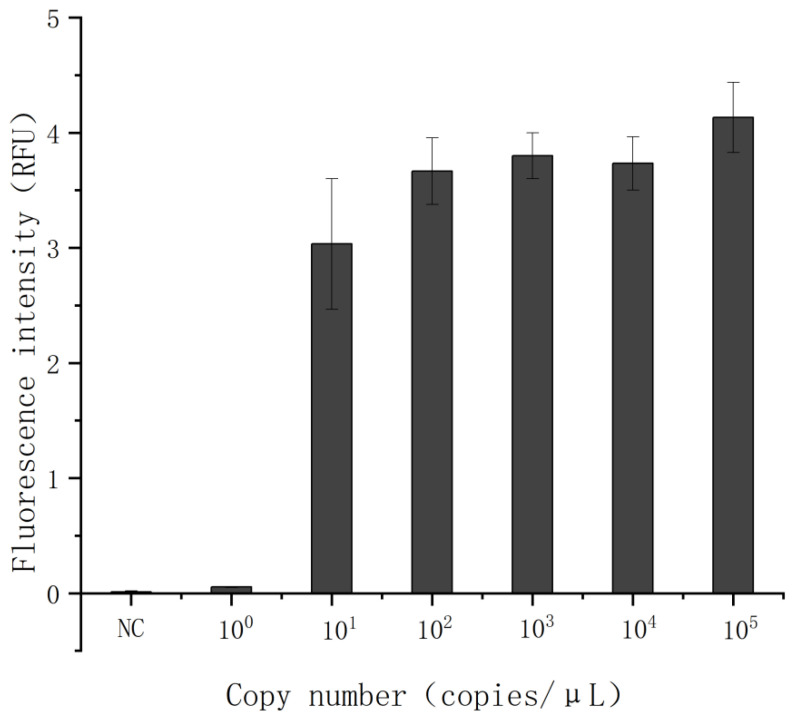
Evaluation of the sensitivity of real-time fluorescent quantitative RT-qPCR. “NC” stands for negative control. The RT-qPCR reaction was carried out with one cycle at 95 °C for 30 s, followed by 40 cycles at 95 °C for 15 s and 40 cycles at 60 °C for 30 s; it was concluded with a melting curve and analyzed in the LC96 PCR system.

The pMD18T-ARVS1 recombinant plasmid standard was serially diluted tenfold to produce template concentrations ranging from 10^0^ to 10^5^ copies/μL. Both the real-time fluorescent RT-RAA assay and the lateral flow detection were performed under optimized conditions. The results showed a consistent detection limit of 10^0^ copies/μL for both methods (Figure 8a–c). Significant differences (*p* < 0.01) were observed between all positive template concentrations and the negative control in the fluorescence-based RT-RAA quantification (Figure 8d). In comparison, RT-qPCR analysis of the same dilution series exhibited a detection limit of 10^1^ copies/μL (Figure 9). In this study, we systematically compared the detection sensitivity of RT-qPCR and the RT-RAA–CRISPR/Cas12a assay, with the results summarized in Table 5. The table enables a clear visual comparison of the performance of both methods across different concentrations of target nucleic acid. Notably, no measurable fluorescence signal was detected by RT-qPCR at 10^0^ copies/μL, and the signal intensity at this concentration showed no statistical significance compared to negative controls (*p* > 0.05). These results collectively demonstrate the superior sensitivity of the optimized RT-RAA/CRISPR–Cas12a detection system under rigorously controlled conditions.

## 4. Discussion

ARV is a genetically diverse pathogen that poses a significant threat to poultry health [36,37]. Economically, it hinders the conversion rate of poultry to feed and even directly leads to death, bringing huge economic losses to the breeding industry. Immunely, it destroys the immune system of poultry, making it more susceptible to secondary infection by other pathogens [8,10], further aggravating the disease. In terms of transmission, ARV can spread rapidly through vertical and horizontal transmission, and it is easy to cause large-scale infection in poultry flocks. Preventing this death is important, but rapid detection at the onset is also crucial [38,39]. However, in some farms with limited equipment, it is difficult to achieve rapid and convenient detection of ARV due to the lack of necessary experimental instruments and technical support. Therefore, rapid, sensitive, and convenient detection is an important condition to help diagnose ARV and reduce losses in poultry farming.

In this study, we developed a novel dual-detection platform based on RT-RAA and CRISPR/Cas12a, which targets the conserved P17 protein-coding region within the ARV S1 gene. This strategy capitalizes on the high sequence conservation of P17, ensuring broad detection capability across diverse ARV strains. The platform simultaneously supports real-time fluorescence and lateral flow strip readouts, providing flexibility for both laboratory and field applications [40,41]. The integration of isothermal amplification with CRISPR/Cas12a collateral cleavage activity achieved a detection sensitivity of 1 copy/μL (Figure 8a–d), surpassing conventional RT-qPCR (10 copies/μL, Figure 9). This enhanced sensitivity stems from the synergistic effects of RT-RAA amplification and Cas12a-mediated secondary signal amplification [42], as demonstrated in our orthogonal optimization experiments (Figure 5 and Figure 6). The specificity of the RT-RAA-CRISPR/Cas12a platform was rigorously validated against non-target avian pathogens, including REV, IBDV, ALV, CAstV, and CAV. Both FQ and LF formats exhibited zero cross-reactivity (Figure 7a-d), which is prone to antibody cross-reactivity. This specificity is attributed to the precise targeting of the conserved P17-coding region (Figure 1) and the sequence-specific recognition of CRISPR/Cas12a. Furthermore, the system’s simplicity, requiring only a portable incubator and visual readout via blue-light analyzers or test strips, eliminates dependency on thermocyclers and skilled personnel, addressing key limitations of RT-qPCR in low-resource environments.

To further enhance the field-deployability of our ARV detection platform, a rapid, equipment-minimized nucleic acid extraction method—thermal lysis—can be employed for on-site sample processing [43,44]. Although this approach enables the release of viral DNA within 10 min without requiring commercial kits, it presents inherent challenges, including reduced DNA purity due to co-purification of host-derived inhibitors and compromised stability. Nevertheless, future work will focus on optimizing a simplified, low-cost sample preparation protocol to achieve seamless integration with our detection assay.

## 5. Conclusions

The RT-RAA-CRISPR/Cas12a platform offers transformative potential for poultry disease management [45]. In China, where poultry production exceeds 17 billion birds annually, early ARV detection can mitigate immunosuppression-induced secondary infections and reduce mortality rates. Field deployment of this method in farms or rural clinics could enable real-time outbreak monitoring, allowing immediate culling or vaccination interventions. Cost analyses from prior studies suggest CRISPR-based diagnostics are 50% cheaper than PCR [46], further enhancing its feasibility for widespread adoption. Additionally, the dual-output design (FQ and LF) accommodates diverse scenarios [47,48]. While this study focused on ARV, the modularity of the RT-RAA-CRISPR/Cas12a system permits adaptation to other avian pathogens by redesigning crRNA and primers. Future studies will validate this platform using clinical samples from geographically diverse ARV strains to assess its robustness against genetic variability.

## Figures and Tables

**Table 1:** Primer sequences of RT-RAA and common PCR.

Primer Name	Sequence (5′-3′)	Size (bp)
RT-RAA1	F-GGACGGATTAACTCAACAGCAGCGAAGAGAA	213
	R-AACGAGGCCGCCACGTTATCAAGCGATGCAG	
RT-RAA2	F-TCAATCCCTTGTTCGTCGATGCTGCGTATG	175
	R-AATAAGAAACCACCACCCGCCGCTAGATAAG	
RT-RAA3	F-TGCGACTGCTGTATTTGGTAAC	233
	R-TGCTTACCAGAACTCAACGCTA	
RT-RAA4	F-CTTTTTCAATCCCTTGTTCGTC	289
	R-TGCTTACCAGAACTCAACGCTA	
S1-PCR	F-GGTGCGACTGCTGTATTTGGTAAC	513
	R-AATGGAACGATAGCGTGTGGG	
qPCR	F-TGCGACTGCTGTATTTGGTAACG	114
	R- CCACCCGCCGCTAGATAAGG	

**Table 2:** crRNA and probe sequences.

Name	Sequence (5′-3′)
crRNA	UAAUUUCUAAGUGUAGAUCAAGCUACGUCACCAUAAUUGC
Lateral Flow (LF) reporter	FAM—TTATT—Biotin
Fluorescent quencher (FQ) reporter	FAM—TTATT—BHQ-1

**Table 3:** Thermocycler Program for RT-qPCR Assay.

Step	Temperature	Time	Cycle
1	95 °C	30 s	1
2	95 °C	5 s	40
	60 °C	30 s	
3		Dissociation Stage	

**Table 4:** Screening of crRNA and Cas12a Concentrations (Data presented as mean ± standard deviation).

		Cas12a Protein Concentration(nM).				
crRNA concentra-tion (nM)		25	50	100	250	500
	5	0.08 ± 0.02	0.1 ± 0.01	0.12 ± 0.01	0.85 ± 0.24	1.07 ± 0.05
	25	0.1 ± 0.01	0.12 ± 0.01	0.29 ± 0.13	0.53 ± 0.04	1.44 ± 0.09
	50	0.12 ± 0.02	0.28 ± 0.06	0.27 ± 0.05	0.98 ± 0.07	1.41 ± 0.06
	100	0.14 ± 0.01	0.28 ± 0.02	0.25 ± 0.06	1.38 ± 0.02	1.63 ± 0.08
	250	0.19 ± 0.02	0.42 ± 0.05	0.44 ± 0.07	2.28 ± 0.07	2.11 ± 0.18
	500	0.18 ± 0.02	0.37 ± 0.03	0.3 ± 0.07	1.83 ± 0.07	2.19 ± 0.08

**Table 5:** Comparison of Sensitivity: RT-qPCR vs. RT-RAA-CRISPR/Cas12a Fluorescence Intensity (RFU).

Copy Number (Copies/μL)	RT-RAA-CRISPR/Cas12a Fluo-Rescence Intensity (RFU)	RT-qPCR Fluo-Rescence Intensity (RFU)
NC	0.00	0.00
10^0^	3.67	0.03
10^1^	3.69	3.11
10^2^	3.75	3.70
10^3^	4.32	3.83
10^4^	4.84	3.75
10^5^	5.33	4.18

## Data Availability

The data presented in this study are available from the corresponding author upon reasonable request.
